# Antioxidant Effects of a Hydroxytyrosol-Based Pharmaceutical Formulation on Body Composition, Metabolic State, and Gene Expression: A Randomized Double-Blinded, Placebo-Controlled Crossover Trial

**DOI:** 10.1155/2017/2473495

**Published:** 2017-08-09

**Authors:** Carmela Colica, Laura Di Renzo, Domenico Trombetta, Antonella Smeriglio, Sergio Bernardini, Giorgia Cioccoloni, Renata Costa de Miranda, Paola Gualtieri, Paola Sinibaldi Salimei, Antonino De Lorenzo

**Affiliations:** ^1^CNR, IBFM UOS of Germaneto, University “Magna Graecia” of Catanzaro, Campus “Salvatore Venuta”, 88100 Germaneto, Catanzaro, Italy; ^2^Section of Clinical Nutrition and Nutrigenomics, Department of Biomedicine and Prevention, University of Rome Tor Vergata, Via Montpellier 1, 00133 Rome, Italy; ^3^Department of Chemical, Biological, Pharmaceutical and Environmental Sciences, University of Messina, Viale F. Stagno d'Alcontres 31, Messina, Italy; ^4^Division of Clinical Biochemistry and Clinical Molecular Biology, University of Rome Tor Vergata, Rome, Italy; ^5^PhD School of Applied Medical-Surgical Sciences, University of Rome Tor Vergata, Via Montpellier 1, 00133 Rome, Italy; ^6^CAPES Scholarship (Proc No. BEX 13264/13-3), CAPES Foundation, Ministry of Education of Brazil, 70040-020 Brasília, DF, Brazil

## Abstract

Hydroxytyrosol (HT) plays a significant role in cardiovascular disease (CVD) protection, and its metabolites are able to protect from the endothelial dysfunction commonly present in atherosclerosis. This randomized double-blinded, placebo-controlled crossover trial determined the effect in healthy volunteers of two gastroresistant capsules containing 15 mg/day of HT, for a 3-week period (HTT). Evaluation of nutritional status, serum metabolites, oxidative stress biomarkers, and gene expression of 9 genes related to oxidative stress, inflammation, and CVDs was performed. Oxidation biomarkers like thiol group (*p* = 0.001), total antioxidant status (TAS) (*p* = 0.001), superoxide dismutase 1 (SOD1) (2^−ΔΔCt^ = 3.7), and plasma concentration of HT (2.83 *μ*g·mL^−1^) were significantly increased, while nitrite (*p* = 0.001), nitrate (*p* = 0.001), and malondialdehyde (MDA) (*p* = 0.02) were drastically reduced after HTT. A significant reduction of body fat mass percentage (*p* = 0.01), suprailiac skinfold (*p* = 0.01), and weight (*p* = 0.04; Δ% = −0.46%) was observed after HTT. This study shows that regular intake of 15 mg/day of HT changed body composition parameters and modulated the antioxidant profile and the expression of inflammation and oxidative stress-related genes. However, it is advisable to personalize HT doses in order to exert its health benefits in CVD prevention and protection of LDL-C particles from oxidative damage. This trial is registered with ClinicalTrials.gov NCT01890070.

## 1. Introduction

Extra virgin olive oil (EVOO) is the main lipid component of Mediterranean diet [1]. The Seven Countries Study, and many other studies [2–4], demonstrated that Mediterranean diet is able to reduce the incidence of chronic degenerative diseases, while the PREDIMED study proved the efficacy of Mediterranean dietary program supplemented with olive oil reduced the incidence of major cardiovascular events due to several changes in pathways involved in cardiometabolic risk [5, 6]. EVOO's ability to improve health conditions is due to several components, like its high content of monounsaturated fatty acids (MUFAs), in particular oleic acid, which is able to improve *α*-linolenic acid (ALA) conversion in longer-chain n-3 polyunsaturated fatty acids (PUFAs), leading to great health benefits on cardiovascular diseases (CDVs) [7]. The Food and Drug Administration (FDA) had recognized olive oil (23 g/day) as a qualified health claim to decrease the risk of coronary heart disease [8].

EVOO supplementation brings several benefits on CVDs, due to its antioxidant, anti-inflammatory, vasodilatory, and antiplatelet aggregation properties [9, 10]. However, the main EVOO cardioprotective effect is attributable to the presence of its phenolic compounds [11]. In fact, European Food Safety Authority (EFSA) scientific opinion has substantiated “health claims” related to specific EVOO phenolic compounds. A daily amount of 5 mg of hydroxytyrosol (3,4 dihydroxyphenylethanol; 3,4-DHPEA or HT) and its derivatives are responsible for the validated health claim “protection of blood lipids from oxidative stress” [12]. HT is the most abundant phenolic compound in EVOO and plays a significant role in CVD protection, and its metabolites are able to protect from endothelial dysfunction commonly present in atherosclerosis [13]. In particular, HT is able to reduce hypercholesterolemia and malondialdehyde (MDA) concentrations [14], inhibit peroxidation of low-density lipoprotein cholesterol (LDL-C) [15], and increase high-density lipoprotein cholesterol (HDL-C) [16, 17]. In vitro and in vivo studies demonstrated that EVOO [18] and HT supplementation [19, 20] also improves antioxidant enzyme activity of superoxide dismutase 1 (SOD1), involved in the pathogenesis of various diseases like atherosclerosis and hypertension [21]. SOD1 is able to catalyze superoxide radical (O_2_^−^) in hydrogen peroxide (H_2_O_2_), which in turn is catalyzed into water through glutathione peroxidase (GSH-Px) and catalase (CAT) [21]. As well as SOD1, GSH-Px, and CAT, several enzymes are involved in the regulation of oxidative stress like macrophage migration inhibitory factor (MIF) [22] and peroxisome proliferator-activated receptor gamma (PPAR*γ*) [23]. Conversely, reactive oxygen species (ROS) have the ability to modulate the expression of different inflammation genes like nuclear factor of kappa light polypeptide gene enhancer in B-cells (NFkB) and consequently chemokine (C-C motif) ligand 2 (CCL2) [24, 25]. A massive ROS amount, particularly radical superoxide, could lead to the a reduction of nitric oxide (NO) bioavailability, one of the main characteristics of CVDs, due to increased peroxynitrite (ONOO^−^) production [26], which seems to be inhibited by HT in vitro [27].

Based on the antioxidant effects in dietary treatment with EVOO or HT supplementation, we hypothesized that 3 weeks administration of 15 mg/day of HT present in a new formulation, with intestinal release, could lead to a change in nutritional status. The first endpoint of this study was to evaluate the body composition parameter changes; the secondary endpoint was to verify the antioxidant status and the glucose and lipid profile. The third endpoint was the evaluation of HT antioxidant effect on gene expression of selected genes belonging to inflammatory and oxidative stress pathways. Therefore, a randomized clinical trial was performed on healthy volunteers in a free dietary regimen.

## 2. Methods

### 2.1. Study Design and Subjects

The study was conducted using a double-blinded, randomized, placebo-controlled crossover design, between December 2015 and April 2016.

**Figure 1:**
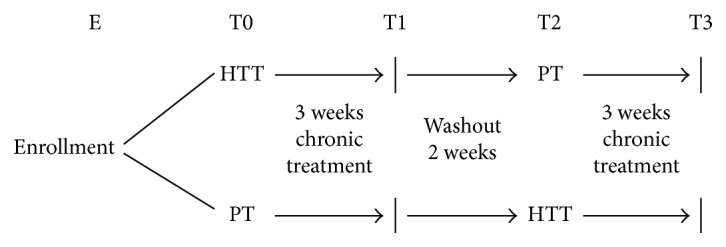
Study design. Clinical trial design: HTT, 15 mg/day hydroxytyrosol treatment; PT, placebo treatment.

Forty subjects were recruited sequentially within a routine medical checkup program at the Section of Clinical Nutrition and Nutrigenomics, Department of Biomedicine and Prevention of the University of Rome Tor Vergata, and twelve of them were excluded from the trial (four subjects did not meet the inclusion criteria, while eight subjects declined to participate). Finally, twenty-eight patients who were between 18 and 65 years old and had a body mass index (BMI) between 18.5 and 29.9 kg/m^2^ respected the inclusion criteria and completed the trial. Due to the different phenotypes of obesity, according to De Lorenzo et al. [28], we selected as eligible for the study only healthy subjects that belong to the normal or overweight range of BMI (18.5 and 29.9 kg/m^2^; total body fata mass percentage (TBFat%) < 30% for women and <25% for men) and subjects who are classified as metabolically healthy obese (TBFat% > 30% for women and >25% for men), without metabolic complications. Eligible patients were randomly divided in two groups (1 : 1 ratio) and took daily note 2 capsules of 7.5 mg/capsule of HT, or n.2 capsules of placebo. HT capsules were administrated 1 time/day, 2 h before lunch in order to ensure gastric transit and intestinal absorption. According to EFSA scientific opinion, we have established to administer 15 mg/die of HT in order to guarantee the achievement of the HT portion set by the authority. A person not involved in the clinical trial carried out the randomization. The study consists of 3 weeks chronic treatment with HT (HTT) or placebo (PT), to ensure complete adhesion of healthy volunteers to the protocol separated by a - week washout period, resulting in a total duration of 8 weeks. At the end of washout, the groups received the other treatment for the same period (Figure 1). At the beginning and at the end of each treatment, body composition evaluation, gene expression, and blood analysis were performed. Subjects were asked to maintain their usual lifestyle habits and to report any illness or abnormality arising during the study.

The primary outcome of this study was the evaluation of nutritional status according body composition changes measured by anthropometry, plicometry, and bioimpedentiometry (BIA), after HTT. The secondary outcome was to asses a panel of blood analysis, on antioxidant status (total antioxidant status (TAS) and lipid peroxidation, expressed as MDA, thiols, nitrites/nitrates), glucose and lipid profile (TC, high-density lipoprotein cholesterol (HDL-C), low-density lipoprotein cholesterol (LDL-C), and triglycerides (Tg)), and plasma and erythrocytes membrane PUFAs.

The third outcome was the evaluation of antioxidant effect of HTT on gene expression of selected genes belonging to inflammatory and oxidative stress pathways: MIF, SOD1, PPAR*γ*, CAT, CCL2, NFkB1, methylenetetrahydrofolate reductase (MTHFR), apolipoprotein E (APOE), and angiotensin I-converting enzyme (ACE).

All participants recruited in the study authorized their participation by reading and signing the informed consent, conducted in accordance with the provisions of the Ethics Committee of Medicine, University of Rome Tor Vergata and with the Helsinki Declaration of 1975 as revised in 1983. Trial registration: NCT01890070.

### 2.2. Exclusion Criteria

The exclusion criteria are as follows: pregnancy; breast-feeding; type 1 and type 2 diabetes; heart failure; endocrine disorders; liver dysfunction; liver, kidney, autoimmune, chronic viral (hepatitis C and B, HIV), and neoplastic diseases; corticosteroid and chronic inflammatory therapy; and participation in other dietary trials.

### 2.3. Sample Size

Insulin value was selected as a parameter to calculate minimum sample size according to de Bock et al. [29]. The minimum sample size was calculated using a two-tailed one-sample Student's *t*-test, considering the following: (i) insulin to be detected between baseline and HTT |*δ*| ≥ 15 *μ*U/mL—1; (ii) SD of the paired differences, SD = 15 *μ*U/mL; and (iii) type I error probability *α* = 0.05 and power 1 − *β* = 0.90. The result was a minimum sample size of 10 per group.

### 2.4. HT and Placebo Capsule Composition

HT capsules were composed of the following: hard gelatin enteric coated capsules (Fenolia™, P&P Farma Srl, Turin, Italy) at 7.5 mg strength of hydroxytyrosol from olive extract (elaVida™, DSM, Heerlen, The Netherlands) in extra virgin organic olive oil vehicle and enteric coated with Eudraguard® natural (Evonik Industries AG, Essen, Germany). Placebo capsule formulation was of the same sample formulation (HT capsules) but without the elaVida extract.

### 2.5. Anthropometric, Bioimpendance Analysis, and Dual-Energy X-Ray Absorptiometry

Waist and hip circumferences were taken using a flexible steel metric tape to the nearest 0.5 cm, according to International Society for the Advancement of Kin anthropometry protocol and National Institute of Health Guidelines. Body weight (kg) was measured to the nearest 0.1 kg, using a technical balance (Invernizzi, Rome, Italy). Waist/hip ratio (WHR) was also evaluated in relation to clinical risk thresholds, that is, WHR > 1 for men and WHR > 0.9 for women. Height (m) was measured to the nearest 0.1 cm using a stadiometer (Invernizzi, Rome, Italy) [30]. BMI was calculated using the formula BMI = body weight/height^2^ (kg/m^2^). Body composition analysis was assessed by BIA phase sensitive system (BIA 101S, Akern/RJL Systems, Florence, Italy) [31], and total body fat mass (TBFat) and total body lean mass (TBLean) were assessed using a dual-energy X-ray absorptiometry (DXA) (i-DXA, GE Medical Systems, Milwaukee, WI, USA) according to the previously described procedures [26, 32].

Fat mass (FM) distribution was determined using a plicometer (Harpenden Callipers) with skinfold measurement in triplicate and collected at four points: triceps, biceps, subscapular, and suprailiac. Skinfold measurements were utilized in Durnin and Womersley's equations to calculate body density, and the Siri formula was subsequently applied to predict FM [33].

### 2.6. Dietary Assessments

Before the beginning of the clinical trial, a validated and adapted dietary questionnaire was self-administered [34]. The food intake before and during the clinical trial was assessed from 3-day diet records completed for two weekdays and one weekend day [35]. The subjects were instructed by a dietitian to record weight and/or measures of all foods and beverages consumed, to use product brand names when recording dietary intake. Photographs of food portion sizes were provided to help estimate the amount of food consumption. Diet records were reviewed as they were turned in to confirm that all written food items were legible and to clarify the amounts of foods consumed. The estimated intake of macronutrients was calculated by using the software Dietosystem®.

### 2.7. Analysis of Blood Samples

#### 2.7.1. Biochemical Analysis

Blood samples were taken after a 12-hour overnight fast. Blood samples were collected in sterile tubes containing EDTA (Vacutainer®) and placed on ice. Plasma was separated by centrifugation (1600 rpm, at 4°C for 10 min), removed, aliquoted, and stored at −80°C. All clinical chemistry analyses, except plasma glucose and serum lipid analysis, were carried out using an ADVIA®1800 Chemistry System (Siemens Healthcare) following standard procedures [36]. Plasma glucose concentrations were measured using the glucose oxidase method and automated glucose analyzer (COBAS INTEGRA 400, Roche Diagnostics, Indianapolis, IN, USA); serum lipid profile components were determined by standard enzymatic colorimetric techniques (Roche143 Modular P800, Roche Diagnostics, Indianapolis, IN, USA). Cardiovascular risk was determined by the following ratios: TC/HDL-C (<3) and LDL-C/HDL-C (<2). Adipocyte dysfunction was evaluated by the lipid accumulation product index (LAP) calculated using the following formulas:

(1) $$\begin{array}{c} \left(Waist\;circumference\;\left[cm\right]\;\;–\;\;65\right)\times Tg\;concentration\;\left[mM\right]\;for\;men,\\ \left(Waist\;circumference\;\left[cm\right]\;\;–\;\;58\right)\times Tg\;concentration\;\left[mM\right]\;for\;women. \end{array}$$

Atherogenic Index of Plasma (AIP) was determined using the following formula:

(2) $$\begin{array}{c} Log\left(\frac{Tg\;concentration\;\left[mg/dL\right]}{HDL}-C\;\left[mg/dL\right]\right). \end{array}$$

All the analyses were performed in duplicate, while the measurements were performed in triplicate.

#### 2.7.2. Antioxidant Status

Total antioxidant status (TAS) and lipid peroxidation (expressed as MDA, thiols, and nitrites/nitrates) were determined using commercially available kits following the manufacturer's instructions. Fluorometric thiol assay kit (MAK151-1KT), MDA assay kit (MAK085-1KT), and nitrite/nitrate assay kit (23479-1KT-F) were purchased from Sigma-Aldrich (Milan, Italy). Total antioxidant status assay kit (615700-1KIT) was purchased from Merck Millipore (Milan, Italy). Oxidized LDL-C (ox-LDL) concentration in plasma was measured by enzyme-linked immunosorbent assay using the mAb-4E6 antibody (Mercodia AB, Uppsala, Sweden).

Total plasma and cell membrane PUFAs were analyzed from venous blood sample and collected in tubes containing EDTA (Vacutainer) and centrifuged (3500 rpm for 5 min). Plasma was removed and stored at −80°C while red cells were washed with saline solution (NaCl 0.9%), collected, and freezed at −80°C. Erythrocyte membrane PUFAs methyl esters were extracted by a modified version of previously reported methods [37]. In plasma, total PUFA methyl esters were evaluated through gas chromatography (GC, model 6890N, Agilent Technologies Italia Spa), coupled to a mass spectrometric detector (MSD, model Agilent 5973 inert, Agilent Technologies Italia Spa). Chromatographic separation was performed on a BPX-70 (70% cyanopropyl polysilphenylensiloxane, SGE Analytical Science, Austin, TX, USA) 30 m capillary column (0.25 mm ID, 0.25 *μ*m film thickness). All the analyses were performed in triplicate.

### 2.8. Sample Collection, RNA Extraction, and Analysis

Blood samples were collected, stabilized in PAX gene blood RNA tubes (Pre AnalytiX Qiagen, Hombrechtikon, Switzerland), and stored at −80°C. Total RNA of each collected sample was purified with PAX gene blood miRNA kit following the manufacturer's instructions (Pre Analytix Qiagen, Hombrechtikon, Switzerland). Total RNA was quantified and assessed for quality by spectrophotometry (Nanodrop, Wilmington, USA) and agarose gel electrophoresis. Specific RT2 profiler PCR arrays (Qiagen, Netherlands) were used for human oxidative stress (PAHS-065ZA, Qiagen, Netherlands) and human inflammation (PAHS-097ZA, Qiagen, Netherlands) pathways. Gene expression of the following 9 genes was analyzed: MIF (GenBank accession note AF469046.1), SOD1 (GenBank accession note AK312116.1), PPAR*γ* (GenBank accession note AB307692.1), CAT (GenBank accession note AY545477.1), CCL2 (GenBank accession note AK311960.1), NFkB1 (GenBank accession note AK1122850.1), methylenetetrahydrofolate reductase (MTHFR) (GenBank accession note AB209113.1), apolipoprotein E (APOE) (GenBank accession note K314898.1), and angiotensin I-converting enzyme (ACE) (GenBank accession note AB208971.1). Each qRT-PCR experiment was performed in triplicate and repeated at least twice, in line with the manufacturer's instructions (Qiagen, Netherlands).

### 2.9. HT Bioavailability Determination

HT was extracted from acidified plasma by solid-phase extraction using an Oasis HLB 1 cc vacuum cartridge 30 mg, 30 *μ*m (Waters, Italy) according to Ruiz-Gutiérrez et al. [38]. The analytical evaluation was performed following Miralles et al. [39] with some modifications using an Agilent HPLC system (1100 series) coupled with a DAD detection and equipped with an autosampler refrigerated at 4°C. The elution gradient consisted of mobile phase (A) H_2_O (0.2% CH_3_COOH, pH 3.1) and (B) CH_3_OH. HT was recognized and quantified at 280 nm based on the supplier's standard, comparing retention time and relative UV-vis spectra (range 200–400 nm). A 500 *μ*g·mL^−1^ HT standard stock solution was prepared using acetic acid 0.2% and methanol 75/25 (*v*/*v*) (pH 3.30). From this solution, freshly working standard solutions from 3 to 100 *μ*g·mL^−1^ were prepared. HT ≥ 98% were purchased from Sigma-Aldrich (Milan, Italy). Glacial acetic acid, phosphoric acid, and methanol were HPLC grade and purchased from Merck (Darmstadt, Germany). All the analysis were performed in triplicate.

### 2.10. Statistical Analysis

Statistical analysis was carried out using IBM SPSS 21.0 for Windows (IBM Corp., Armonk, NY, USA). After the Shapiro-Wilk test, a paired *t*-test or a nonparametric Wilcoxon test was performed to evaluate differences before and after HTT. In all statistical tests performed, the null hypothesis (no effect) was rejected at the 0.05 level of probability.

Correlation was performed using a parametric (Pearson) or nonparametric test (Spearman's rank correlation). Data analysis of quantitative real-time PCR: the value used to plot relative gene expression was determined using the expression fold change (FC) = 2^−ΔΔCt^, using *β*-actin (ACTB) as housekeeping gene. Only genes with a FC > 2 were selected and referred to as “differentially expressed genes,” with a *p* value < 0.05 taken to indicate statistical significance.

A test for carry-over effects according to Kenward and Jones [40] was applied. No carry-over effects between the two treatment periods could be observed. All analyses are presented on a per-protocol basis. Of the 40 participants enrolled, 28 subjects concluded the study and for these, complete data sets were available.

## 3. Results

### 3.1. Subjects Characteristics

Of the forty subjects enrolled, twelve of them were excluded from the trial (four subjects did not meet the inclusion criteria, while eight subjects declined to participate). Finally, twenty-eight patients completed the trial (Figure 1). Any changes to trial outcomes after the trial commenced occurred. The average age of subjects was 32.00 ± 12.22 years, where 57.1% were female and 42.9% were male (Table 1). The sample divided for sex presented differences for the following parameters: height, waist/hip circumference ratio, TBW, ICW, ECW, TBFat%, TBLean (kg), triceps, and FM% skinfold (*p* = 0.001); biceps skinfold (*p* = 0.01); sovrailiac skinfold, phase angle, and TBFat (kg) (*p* = 0.05). No changes for bioclinical analysis were observed. The sample divided for age presented differences for the following parameters: waist/hip circumferences ratio and ECW (*p* = 0.001); waist circumference and TBW (*p* = 0.01); height (*p* = 0.02); TBLean (kg) (*p* = 0.03); diastolic blood pressure (DBP) and Tg (*p* = 0.04); and weight (*p* = 0.05). No changes for other bioclinical analysis were observed.

According to the dietary questionnaires, 78.6% and 85.7% of subjects presented an adequate food frequency and food habits, respectively, at the same time 21.4% and 14.3% of subjects presented a discrete food frequency and food habits.

### 3.2. Food Intake Analysis

Concerning food intake analysis, there were not significant differences between HTT and PT. Total energy intake were 1925.33 kcal and 1602.57 kcal (*p* = 0.44) during HTT and PT, respectively. Carbohydrate intake was 250.46 g and 225.64 g (*p* = 0.67), protein intake was 76.04 g and 72.20 g (*p* = 0.58), and lipid consumption was 69.60 g and 45.70 g (*p* = 0.21) during HTT and PT, respectively.

### 3.3. Biochemical Data

After HTT, a significant decrease in nitrite (*p* = 0.001), nitrate (*p* = 0.001), and MDA (*p* = 0.02), as well as an increase in thiol groups (*p* = 0.001) and TAS values (*p* = 0.001) (Table 2), were obtained. These results matched the significant increase in HT bioavailability (2.83 *μ*g·mL^−1^; min–max: 2.25–3.50). No significant changes were observed in ox-LDL-C concentration (Table 2). Moreover, no significant changes were observed after HTT in Tg, TC, and HDL-C (Table 2), as well as in plasma arachidonic acid (AA)/eicosapentaenoic acid (EPA) and AA/docosahexaenoic (DHA) ratios, PUFAs erythrocytes membrane, and plasma concentrations (Table 3). After HTT, a positive correlation between TC and LDL-C was observed (*ρ* = 0.94; *p* = 0.001) (Table 4(b). Furthermore, a positive correlation between nitrite and HT bioavailability (*ρ* = 0.84; *p* = 0.02) was observed after HTT (Table 4(b)). On the other hand, a significant positive correlation between MDA and nitrite concentration (*ρ* = 0.76; *p* = 0.04) at baseline was observed. This correlation was not kept after treatment (Table 4(a)).

### 3.4. Gene Expression Data

**Figure 2:**
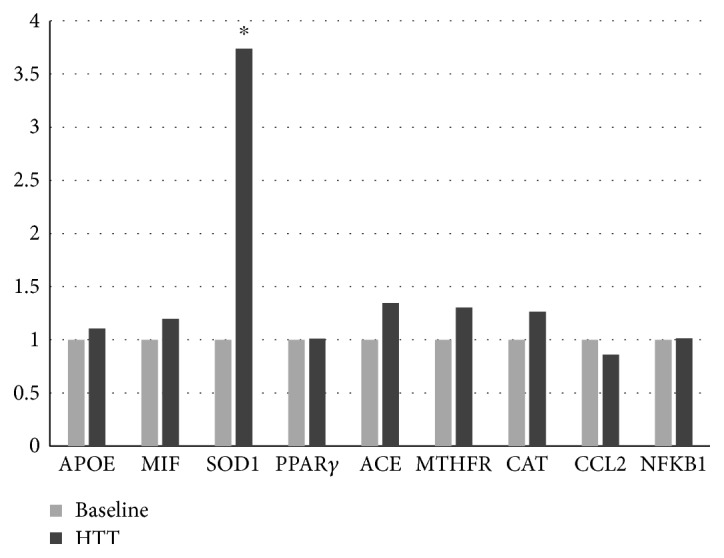
Gene expression on baseline and after HTT. Different levels of fold change of genes analyzed baseline (B) and HTT; ^∗^*p* < 0.05. APOE: apolipoprotein E; MIF: macrophage migration inhibitory factor; SOD1: superoxide dismutase 1; PPAR*γ*: peroxisome proliferator-activated receptor gamma; ACE: angiotensin I-converting enzyme; MTFHR: methylenetetrahydrofolate reductase; CAT: catalase; CCL2: chemokine (C-C motif) ligand 2; NFkB1: nuclear factor of kappa light polypeptide gene enhancer in B-cells 1.

Significant upregulation of SOD1, with a fold change exceeding the threshold set at 2, was observed (2^−ΔΔCt^ = 3.7) after HTT (Figure 2). At baseline (T0), correlation analysis showed a significant negative correlation (*ρ* = −0.87; *p* = 0.01) between SOD1 gene expression (2^−ΔCt^ = 0.01) and the concentration of circulating nitrites (Table 3). No other significant gene expression changes were observed.

### 3.5. Body Composition Data

After HTT, a significant reduction in weight (*p* = 0.04; Δ% = −0.46%), FM% (*p* = 0.01; Δ% = −3.52%) and suprailiac skinfold (*p* = 0.01; Δ% = −6.1%) were observed. BIA showed a significant increase in phase angle both in HTT (*p* = 0.01; Δ% = 7.23%) and PT (*p* = 0.02; Δ% = −2.45%) in reduction of extracellular water (ECW) for both treatments (HTT: *p* = 0.001; Δ% = −5.44%; PT: *p* = 0.02; Δ% = 1.69%) (Table 5).

## 4. Discussion

EVOO, used in the traditional Mediterranean diet, contains phenolic compounds that have several beneficial effects on lipoproteins, inflammatory markers, oxidative damage, cellular and platelet functions, bone integrity, and antimicrobial activity [41–43]. Moreover, food industries have manifested great interest in polyphenol bioavailability and in “functional food” based on specific polyphenol content [44, 45]. The EFSA established for the EVOO polyphenol the health claim “protection of blood lipids from oxidative stress.” This health claim is valid only if the daily intake of HT and its derivatives is at least 5 mg/die, in order to have a protection of LDL particles from oxidative damage [12]. In the present study, the administration of 15 mg/die of HT was established in order to guarantee the achievement of recommended dose.

HT antioxidant activity is related to the high bioavailability and elevated absorption capacity found to be essential for pharmacokinetic properties. A stable resonance structure is produced by the catecholic structure of HT, which is able to scavenge the peroxyl radicals and interrupt a peroxidative chain reaction [16]. Compared to intravenous administration, the ingestion of HT, conveyed in EVOO, is more effective in terms of bioavailability [46].

Vissers et al. [47] demonstrated that healthy volunteers and ileostomy patients absorb EVOO polyphenols equally, concluding that most phenols ingested are absorbed by the small intestine. In our study, plasma HT concentration showed a significant increase after intervention. We observed the presence of 2.83 *μ*g·mL^−1^ of HT in plasma proving that the gastroresistant formulation adopted allows the HT, as well as other EVOO polyphenols, to be released in the intestine without suffering degradation in the stomach and showing that the type of vehicle used allowed the HT to remain for a longer period in the plasma compartment.

The sample divided for sex presented some significant differences for height and body composition parameters, at the same time the sample divided for age presented significant differences for height, body composition parameters, and DBP and Tg concentrations. However, parameters which resulted different in the analysis are perfectly in line with physiological differences between males and females and physiological adaptation for different age [48–50].

In line with Anderson-Vasquez et al. [51], we observed a significant reduction in weight and suprailiac skinfold and FM% after HTT. FM accumulation could lead to several metabolic changes which incremented CVD risk [52]. The endocrine role of adipose tissue, with the related release of inflammatory cytokines, reduces the oxidative damage response and at the same time increases oxidative stress [53]. Body weight and TBFat reduction determines the improvement of the response to oxidative processes. In as much as body weight reduction was observed only after HTT in a free dietary regimen, without significant changes in energy intake during all the time of the study, the antioxidant effect is dependent on HTT, in a vicious cycle. At the same time, we reported a significant increase in phase angle, with a substantial reduction in ECW in liters during both HTT and PT, results that are not attributable to a specific treatment.

This study has highlighted the significant effect of a daily intake of 15 mg/day HT supplementation compared to placebo on oxidation markers, demonstrating the possibility to reduce cardiovascular risk. Human oxLDL-C are linked to atherosclerosis, coronary heart disease, and coronary events [54]. oxLDL-C can be bound by phenolic compounds present in plasma, which have a peroxyl scavenging activity in the arterial intima [55], and their concentrations decrease with increased consumption of phenolic compounds. Furthermore, Briante et al. [56] demonstrated that HT exerts its antioxidant effects only at high concentrations. However, results on direct correlations between oxLDL-C and phenolic compound concentrations are conflicting [57]. In contrast to other studies [16, 58, 59], in which it was reported that the intake of EVOO, especially with the high content of secoiridoidic derivatives, decreased the concentration of oxLDL-C, TC, Tg, LDL, and LDL/TC ratio, we did not observe any significant change in these parameters after HTT. The highlighted effects of the other studies by may depend on the simultaneous intake of different EVOO polyphenols, for a long period, while the amount present in the pharmaceutical formulation used may not be sufficient to modulate lipid profile in 3 weeks treatment. Moreover, previous studies [55, 59] have used HT concentrations based on body weight, which result higher than the amount administrated in this study. Therefore, our data suggest a personalized dose of HT.

Conversely, a significant increase of thiols and TAS values, as well as the reduction of nitrite, nitrate, and MDA concentrations, was shown in this study. In general, TAS is a reflection of oxidative stress, and growing TAS concentration after HTT indicates that HT supplementation leads to the improvement of oxidant activity. Thiols are organic compounds that exist in vivo in three forms, which include the free thiol, homodisulfides, and heterodisulfides, and they are the most represented category of the total body antioxidants, playing a pivotal role in defense against ROS [60] representing a marker of CVDs, especially for atherosclerotic plaque, thickness of intima-media, and vasodilatation of endothelium [61]. The increased thiols concentration after HTT could implicate a prominent role of the phenolic compound in the maintenance of redox balance, probably due to its antioxidant activity, which protects endogenous antioxidant molecules from oxidation. At the same time, MDA concentration indicates the in vivo peroxidation of PUFAs and is considered as an atherogenic factor per se [62]. In accordance to Katsarou et al. [17], we noticed a significant reduction of MDA concentration after HTT, which enforce the idea that HT is able to protect cells membrane and other molecules from oxidation and to reduce the atherosclerosis risk. Furthermore, a positive correlation between MDA and nitrite concentrations was found at baseline (T0), while this was not observed after HTT, proving that the antioxidant effect of HT prevents lipid oxidation. In contrast to González-Santiago et al. [63], we did not notice significant changing neither on lipid profile, except for LDL concentrations, nor in plasma and erythrocyte membrane PUFAs, suggesting that HT supplementation in healthy subjects, as expected, does not exert any change in lipid profile, which instead is modulated by MUFAs contained in EVOO, in accordance with Wahle et al. [7]. In healthy subjects, LDL-C concentrations under 150 mg/dL are considered normal values. The increased LDL-C concentrations that we observed after HTT and PT, although significant, remain normal values and, at the same time, determined the slight, and not significant, increase of TC observed after treatments, as demonstrated by the positive correlation between TC and LDL-C. Furthermore, the significant increase of LDL-C concentrations observed in both HTT and PT, making this changing independent from the treatment. For all these reasons, the increasing LDL-C concentration could be not correlated to the increase of atherogenic risk.

Furthermore, this study suggests a pivotal role of HT in the nitrite/nitrate management. In vivo and in vitro studies observed that some EVOO compounds are able to exert scavenging activity proprieties on peroxinitrites and nitrites [27, 64]. Our data indicate that HT determines a critical reduction of nitrite and nitrate concentrations in plasma. The possible explanation of this effect could be found in the increasing expression of SOD1 related to HT supplementation, a hypothesis that is in line with Yao et al.'s results [20].

Moreover, at baseline, a significant negative correlation between SOD1 gene expression and the concentration of circulating nitrites emerged. Since SOD1 decreases the amount of peroxynitrites, we presume that HT caused the reduction in this radical species, associated with the increase of SOD1 expression. In fact, we did not observe any correlation between SOD1 expression and nitrite concentration after HTT. As plasma nitrite concentration is a marker of NO generation [65], and the reduction of NO bioavailability is related to endothelial dysfunction, which in turn is associated with the atherosclerosis and CVD onset, our results suggest another possible mechanism of action of HT in CVD prevention in accordance with Zrelli et al. [66]. Furthermore, compared to a reduction of circulating nitrite concentrations after HTT, it was observed that HT bioavailability correlates positively with nitrites (*ρ* = 0.94; *p* = 0.001), suggesting likely the scavenging activity of HT on peroxinitrites, which in turn are commonly transformed into nitrites [16, 67, 68]. All genomic and metabolomics results reflect the high grade of bioavailability of HT supplements.

These findings support the role of HT supplementation in the gene-mediated effects in correlation with the reduction of biomarkers of oxidative stress and improved body composition, in the absence of a specific dietary regime.

Based on our results, the HT consumption of 15 mg/die would be advisable in relation to the reduction of oxidative stress and reduction of cardiovascular risk due to body composition, lipid, and plasma antioxidant profile improvement. Conversely, HT supplementation used in our study, perhaps, is not sufficient to exert antioxidant effects on oxLDL-C.

Unfortunately, the limits of this study were the small number of enrolled subjects and short duration of chronic treatment. However, the calculated sample size has been chosen regarding previous nutrigenomic studies in clinical trials [69, 70]. Since, on the basis of literature results, it has been demonstrated that a 6-week dietary treatment with 50 mL/day of EVOO, which contains an average of 2 mg/L of HT, exerts a positive role on the lipid profile [18], we considered a 3-week treatment period with 15 mg/day of HT, sufficient to assess the efficacy of the treatment itself. Furthermore, a 3-week treatment period was chosen in order to ensure complete adhesion of healthy volunteers to clinical trial of 8 weeks of duration. However, according to our results, 3 weeks of treatment is not sufficient to draw a firm conclusion about HT efficacy. A longer period with cycles of two months of HTT could be helpful.

## 5. Conclusion

In this work, we observed how 15 mg/die of HT consumption could exert positive effects on human health reducing oxidative stress and cardiovascular risk, and improving lipid and plasma antioxidant profile, although this daily amount of HT does not seem to produce any positive effects on oxLDL-C. Set the minimum supplementation of 5 mg/die of HT recommended by EFSA, these results suggest a necessary personalization of HT doses in order to exert its health benefits in CVD prevention and protection of LDL-C particles from oxidative damage. However, further clinical trials are needed on a larger population over a longer period to increase knowledge about therapeutic mechanisms and ensure its efficacy and safety.

## Figures and Tables

**Table 1:** Enrollment clinical and anthropometric characteristics of healthy volunteers.

Parameter		Frequency	
(*n* = 28)		(*n*; %)	
Gender	Male	(*n* = 12; 42.9%)	
	Female	(*n* = 16; 57.1%)	
Body composition phenotype	NWL	(*n* = 12; 42.9%)	
	MHO	(*n* = 16; 57.1%)	
		Mean ± SD	Median (min–max)
(*n* = 28)	(*n* = 28)
Age (years)		32.00 ± 12.22	27.00 (23.00–64.00)
SBP (mmHg)		119.31 ± 7.79	120.00 (100.00–130.00)
DBP (mmHg)		74.46 ± 6.65	76.00 (60.00–80.00)
TC (mg/dL)		156.54 ± 14.36	160.00 (138.00–184.00)
HDL-C (mg/dL)		61.92 ± 4.57	63.00 (54.00–69.00)
LDL-C (mg/dL)		88.69 ± 13.23	86.00 (63.00–110.00)
Tg (mg/dL)		67.77 ± 32.46	58.00 (32.00–134.00)
Glycemia (mg/dL)		72.46 ± 5.62	72.00 (62.00–79.00)
Insulin (*μ*U/mL)		7.58 ± 4.09	6.81 (3.32–17.68)
Height (cm)		165.68 ± 9.29	163.80 (151.50–183.60)
Weight (kg)		66.61 ± 10.06	69.00 (51.10–80.90)
BMI (kg/m^2^)		23.62 ± 2.47	23.46 (20.20–28.00)
Waist circumference (cm)		76.15 ± 6.97	76.80 (66.00–90.40)
Hip circumference (cm)		99.12 ± 8.04	97.90 (89.00–116.00)
Waist/hip ratio		0.77 ± 0.06	0.77 (0.69–0.91)
Biceps skinfold (mm)		7.21 ± 4.37	6.90 (2.20–15.13)
Triceps skinfold (mm)		16.68 ± 6.61	16.80 (7.40–26.50)
Subscapular skinfold (mm)		13.55 ± 4.67	13.80 (6.50–21.90)
Suprailiac skinfold (mm)		12.26 ± 6.14	9.87 (5.40–25.60)
FM% (skinfolds)		24.92 ± 8.08	27.29 (12.74–39.05)
TBW (L)		36.13 ± 6.76	34.00 (27.90–46.40)
ECW (L)		16.81 ± 2.76	15.80 (12.50–21.60)
ICW (L)		19.31 ± 4.40	18.20 (13.20–26.40)
Phase angle		5.81 ± 0.78	5.80 (4.40–7.00)
TBFat% (DXA)		28.26 ± 10.43	30.00 (11.10–44.90)
TBFat (kg)		18.60 ± 7.84	16.64 (7.84–36.13)
TBLean (kg)		45.14 ± 10.80	42.45 (30.99–62.62)
Energy intake (kcal)		1717.48 ± 546.09	1750.32 (1107.20–2585.01)
Carbohydrates (g)		245.89 ± 80.09	234.82 (152.79–413.19)
Protein (g)		76.30 ± 19.95	76.79 (47.37–119.97)
Lipids (g)		54.11 ± 26.41	42.94 (18.78–95.88)

All results were expressed as mean ± standard deviation (SD) (*number of replicates* = 3) and median (minimum and maximum). SBP: systolic blood pressure; DBP: diastolic blood pressure; TC: total cholesterol; HDL-C: high-density lipoprotein cholesterol; LDL-C: low-density lipoprotein cholesterol; Tg: triglycerides; BMI: body mass index; FM: fat mass; TBW: total body water; ECW: extracellular water; ICW: intracellular water; TBFat: total body fat; TBLean: total body lean.

**Table 2:** Biochemical analysis.

Parameter	Baseline (T0) Mean ± SD (*n* = 28)	HTT Mean ± SD (*n* = 28)	*p*	Baseline (T2) Mean ± SD (*n* = 28)	PT Mean ± SD (*n* = 28)	*p*
TC (mg/dL)	155.79 ± 12.19	163.07 ± 18.74	0.11	161.71 ± 18.79	167.75 ± 16.20	0.06
HDL-C (mg/dL)	61.14 ± 9.43	62.21 ± 7.75	0.64	64.18 ± 7.85	64.31 ± 9.19	0.49
Tg (mg/dL)	64.57 ± 29.92	60.71 ± 22.04	0.30	71.18 ± 38.02	81.25 ± 56.74	0.65
LDL-C (mg/dL)	88.14 ± 10.60	93.71 ± 16.38	0.02^∗^	90.82 ± 14.83	95.06 ± 13.39	0.01^∗^
Glycemia (mg/dL)	79.86 ± 8.70	77.21 ± 8.67	0.43	80.1 ± 6.70	76.23 ± 7.77	0.43
Insulin (*μ*U/mL)	6.51 ± 3.00	6.78 ± 2.79	0.52	6.26 ± 2.88	6.48 ± 2.34	0.41
oxLDL-C (U/L)	40.18 ± 6.66	41.32 ± 6.63	0.52	40.98 ± 9.75	44.11 ± 9.32	0.24
TC/HDL-C	2.59 ± 0.35	2.65 ± 0.42	0.97	2.28 ± 0.39	2.06 ± 0.44	0.08
TC/LDL-C	1.78 ± 0.19	1.76 ± 0.17	0.59	1.98 ± 0.29	1.96 ± 0.17	0.23
LAP	10.64 ± 3.70	11.21 ± 4.44	0.36	10.56 ± 3.80	10.27 ± 3.46	0.22
AIP	−0.01 ± 0.22	−0.04 ± 0.19	0.45	−0.01 ± 0.14	−0.01 ± 0.18	0.99
Thiols (*μ*M)	23.04 ± 2.90	27.06 ± 2.51^§^	0.001^∗^	22.90 ± 2.54	23.45 ± 2.08	0.32
Nitrites (*μ*M)	6.90 ± 3.17	2.77 ± 1.86^§^	0.001^∗^	6.59 ± 2.97	5.87 ± 1.79	0.56
Nitrates (*μ*M)	76.72 ± 60.89	46.04 ± 20.00^a,§^	0.001^∗^	75.84 ± 58.63	72.05 ± 61.00^a^	0.77
MDA (*μ*M)	0.44 ± 0.27	0.26 ± 0.23^a,∗^	0.02^∗^	0.49 ± 0.12	0.45 ± 0.33^a^	0.92

All parameters were evaluated before and after HTT. All results were expressed as mean ± standard deviation (SD) (*number of replicates* = 3). Statistical significance attributed to results with ^∗^*p* < 0.05 (between HTT and baseline (T0) or between PT and baseline (T2)) and ^§^*P* < 0.01 (between HTT and baseline (T0) or between PT and baseline (T2)) after parametric test (Student *t*-test) or ^a^nonparametric test (Wilcoxon-Mann–Whitney). HTT: 15 mg/day hydroxytyrosol treatment; PT: placebo treatment; TC: total cholesterol; HDL-C: high-density lipoprotein cholesterol; Tg: triglycerides; LDL-C: low-density lipoprotein cholesterol; TAS: total antioxidant status; oxLDL-C: oxidized low-density lipoprotein; LAP: lipid accumulation product; AIP: atherogenic index of plasma; MDA: malondialdehyde.

**Table 3:** Plasmatic and erythrocyte membrane PUFA concentrations after HTT and PT.

PUFAs	Parameter	Baseline (T0)	HTT	*p*	Baseline (T2)	PT	*p*
		Mean ± SD (*n* = 28)	Mean ± SD (*n* = 28)		Mean ± SD (*n* = 28)	Mean ± SD (*n* = 28)	
Plasmatic	AA/EPA	24.33 ± 29.51	21.47 ± 20.15	0.40	19.18 ± 29.15	17.39 ± 14.44	0.71
Plasmatic	AA/DHA	2.28 ± 0.77	2.43 ± 0.70	0.06	2.29 ± 0.62	2.46 ± 0.83	0.22
Erythrocytes membrane	AA/EPA	60.60 ± 87.20	54.24 ± 76.75	0.12	51.60 ± 74.65	50.26 ± 63.03	0.77
Erythrocytes membrane	AA/DHA	3.51 ± 0.87	3.39 ± 0.78	0.07	3.40 ± 0.81	3.46 ± 0.99	0.56

All parameters were evaluated before and after HTT. All results were expressed as mean ± standard deviation (SD) (*number of replicates* = 3). PT: placebo treatment; HTT: 15 mg/day hydroxytyrosol treatment; AA: arachidonic acid; EPA: eicosapentaenoic acid; DHA: docosahexaenoic acid.

**Table:** (a) Bioclinical and gene expression analysis at Baseline

Baseline		Glycemia (mg/dL)	Insulin (U/mL)	TC (mg/dL)	HDL-C (mg/dL)	Tg (mg/dL)	LDL-C (mg/dL)	TAS (mM)	Thiols (*μ*M)	Nitrites (*μ*M)	Nitrates (*μ*M)	MDA (*μ*M)	SOD1
Glycemia (mg/dL)	*ρ*		−0.37	−0.33	−0.56	−0.45	0.54	0.52	−0.59	0.33	0.21	0.58	−0.20
	*p*		0.42	0.47	0.19	0.31	0.21	0.24	0.16	0.47	0.64	0.17	0.66
Insulin (U/mL)	*ρ*			−0.08	−0.30	0.67	−0.26	−0.61	0.14	0.46	−0.34	0.23	−0.15
	*p*			0.86	0.51	0.10	0.57	0.15	0.76	0.30	0.46	0.62	0.75
TC (mg/dL)	*ρ*				0.52	0.17	0.55	−0.21	0.59	−0.21	−0.26	−0.40	−0.10
	*p*				0.23	0.71	0.20	0.65	0.26	0.65	0.58	0.38	0.83
HDL-C (mg/dL)	*ρ*					−0.29	−0.13	0.21	0.34	−0.61	0.01	−0.49	0.28
	*p*					0.52	0.78	0.65	0.45	0.14	0.98	0.26	0.54
Tg (mg/dL)	*ρ*						−0.25	−0.99	0.65	0.39	−0.19	0.14	−0.35
	*p*						0.59	0.001^∗^	0.11	0.39	0.68	0.77	0.45
LDL-C (mg/dL)	*ρ*							0.26	0.13	0.001	−0.31	−0.03	−0.04
	*p*							0.57	0.79	0.99	0.50	0.94	0.93
TAS (mM)	*ρ*								−0.71	−0.25	0.26	−0.04	0.24
	*p*								0.07	0.59	0.58	0.94	0.61
Thiols (*μ*M)	*ρ*									−0.15	−0.28	−0.35	−0.13
	*p*									0.76	0.55	0.44	0.78
Nitrites (*μ*M)	*ρ*										0.49	0.76	−0.87
	*p*										0.26	0.05^∗^	0.01^∗^
Nitrates (*μ*M)	*ρ*											0.43	−0.70
	*p*											0.34	0.08
MDA (*μ*M)	*ρ*												−0.61
	*p*												0.14

Analysis was conducted using Pearson or Spearman's rank correlation coefficient *ρ*. Statistical significance attributed to results with ^∗^*p* < 0.05. TC: total cholesterol; HDL-C: high-density lipoprotein cholesterol; Tg: triglycerides; LDL-C: low-density lipoprotein cholesterol; TAS: total antioxidant status; SOD1: superoxide dismutase 1.

**Table:** (b) Bioclinical and gene expression analysis after HTT

HTT		Glycemia (mg/dL)	Insulin (U/mL)	TC (mg/dL)	HDL-C (mg/dL)	Tg (mg/dL)	LDL-C (mg/dL)	HT (mg/mL)	TAS (mM)	Thiols (*μ*M)	Nitrites (*μ*M)	Nitrates (*μ*M)	MDA (*μ*M)	SOD1
Glycemia (mg/dL)	*ρ*		−0.46	0.54	−0.68	−0.64	0.73	−0.11	0.64	−0.32	0.41	−0.43	0.14	−0.36
	*p*		0.29	0.21	0.09	0.12	0.07	0.81	0.12	0.48	0.36	0.34	0.77	0.43
Insulin (U/mL)	*ρ*			−0.10	−0.02	0.58	−0.16	0.53	−0.51	−0.25	0.15	0.65	−0.13	0.32
	*p*			0.83	0.97	0.17	0.73	0.22	0.25	0.59	0.76	0.11	0.78	0.48
TC (mg/dL)	*ρ*				−0.07	−0.02	0.94	0.44	−0.04	−0.13	0.68	−0.71	−0.02	−0.29
	*p*				0.88	0.96	0.001^∗^	0.32	0.94	0.78	0.09	0.07	0.97	0.53
HDL-C (mg/dL)	*ρ*					0.23	−0.39	0.17	−0.32	0.32	−0.15	−0.17	−0.25	0.21
	*p*					0.62	0.39	0.71	0.48	0.48	0.75	0.72	0.59	0.65
Tg (mg/dL)	*ρ*						−0.08	0.41	−0.98	0.46	0.08	0.19	−0.35	−0.05
	*p*						0.87	0.37	0.001^∗^	0.30	0.86	0.69	0.44	0.92
LDL-C (mg/dL)	*ρ*							0.36	0.06	−0.10	0.71	−0.67	0.01	−0.45
	*p*							0.43	0.90	0.82	0.07	0.10	0.99	0.31
HT (mg/mL)	*ρ*								−0.34	0.26	0.84	−0.17	−0.47	−0.46
	*p*								0.45	0.57	0.02^∗^	0.71	0.29	0.30
TAS (mM)	*ρ*									−0.44	−0.03	−0.12	0.37	−0.02
	*p*									0.32	0.94	0.79	0.42	0.97
Thiols (*μ*M)	*ρ*										0.22	−0.34	−0.57	−0.70
	*p*										0.64	0.46	0.18	0.08
Nitrites (*μ*M)	*ρ*											−0.46	−0.47	−0.71
	*p*											0.30	0.28	0.07
Nitrates (*μ*M)	*ρ*												−0.02	0.56
	*p*												0.96	0.19
MDA (*μ*M)	*Ρ*													0.46
	*p*													0.30

Analysis was conducted using Pearson or Spearman's rank correlation coefficient *ρ*. Statistical significance attributed to results with ^∗^*p* < 0.05. HTT: hydroxytyrosol treatment; TC: total cholesterol; HDL-C: high-density lipoprotein cholesterol; Tg: triglycerides; LDL-C: low-density lipoprotein cholesterol; TAS: total antioxidant status; SOD1: superoxide dismutase 1.

**Table 5:** Body composition parameters.

Parameter	Baseline (T0) Mean ± SD (*n* = 28)	HTT Mean ± SD (*n* = 28)	*p*	Baseline (T2) Mean ± SD (*n* = 28)	PT Mean ± SD (*n* = 28)	*p*
Weight (kg)	67.35 ± 9.77	67.04 ± 9.98	0.04^∗^	66.74 ± 9.68	67.25 ± 9.91	0.52
BMI (kg/m^2^)	24.39 ± 3.39	24.27 ± 3.51	0.05	24.12 ± 3.33	24.37 ± 3.11	0.94
Waist circumference (cm)	77.24 ± 7.04	78.14 ± 6.93	0.16	76.78 ± 7.15	78.28 ± 9.07	0.09
Hip circumference (cm)	99.07 ± 7.67	98.92 ± 8.34	0.82	98.41 ± 7.92	99.21 ± 7.29	0.47
Waist/hip ratio	0.78 ± 0.07	0.79 ± 0.06	0.13	0.78 ± 0.06	0.78 ± 0.06	0.87
Biceps skinfold (mm)	5.99 ± 2.57	5.44 ± 2.57	0.07	5.57 ± 4.39	5.87 ± 2.49	0.13
Triceps skinfold (mm)	15.93 ± 6.36	14.83 ± 8.02	0.13	15.74 ± 7.16	16.01 ± 7.87	0.58
Subscapular skinfold (mm)	13.39 ± 3.89	12.89 ± 3.69	0.17	13.48 ± 4.65	14.26 ± 5.25	0.66
Suprailiac skinfold (mm)	12.38 ± 5.25	11.63 ± 5.13	0.01^∗^	12.66 ± 6.01	12.60 ± 5.68	0.83
FM% (skinfolds)	24.30 ± 7.29	23.44 ± 7.96	0.01^∗^	23.95 ± 7.14	23.73 ± 8.05	0.13
TBW (L)	38.03 ± 7.25	38.01 ± 7.43	0.98	37.11 ± 6.87	37.23 ± 6.99	0.06
ECW (L)	17.58 ± 8.35	16.62 ± 3.12	0.001^∗^	16.75 ± 2.72	16.34 ± 2.88	0.02^∗^
ICW (L)	20.45 ± 4.91	21.39 ± 4.79	0.14	20.36 ± 4.66	20.18 ± 4.91	0.10
Phase angle	6.02 ± 0.72	6.46 ± 0.87	0.01^∗^	6.51 ± 0.88	6.62 ± 0.85	0.02^∗^

All parameters were evaluated before and after HTT. All results are expressed as mean ± standard deviation (SD) (*number of replicates* = 3). Statistical significance attributed to results with ^∗^*p* < 0.05 (between HTT and baseline (T0) or between PT and baseline (T2)) after parametric test (Student *t*-test). PT: placebo treatment; HTT: 15 mg/day hydroxytyrosol treatment; BMI: body mass index; FM: fat mass; TBW: total body water; ECW: extracellular water; ICW: intracellular water.

## Abbreviations

HT: Hydroxytyrosol
EVOO: Extra virgin olive oil
CVDs: Cardiovascular diseases
HTT: Hydroxytyrosol treatment
TAS: Total antioxidant status
SOD1: Superoxide dismutase 1
MDA: Malondialdehyde
LDL-C: Low-density lipoprotein cholesterol
MUFAs: Monounsaturated fatty acids
ALA: *α*-Linolenic acid
PUFAs: Polyunsaturated fatty acids
FDA: Food and Drug Administration
EFSA: European Food Safety Authority
HDL-C: High-density lipoprotein cholesterol
GSH-Px: Glutathione peroxidase
CAT: Catalase
MIF: Migration inhibitory factor
PPAR*γ*: Peroxisome proliferator-activated receptor gamma
ROS: Reactive oxygen species
NO: Nitric oxide
ONOO: Peroxynitrite
BIA: Bioimpedentiometry
PT: Placebo
HIV: Human Immunodeficiency Virus
BMI: Body mass index
TC: Total cholesterol
RNA: Ribonucleic acid
AA: Arachidonic acid
EPA: Eicosapentaenoic acid
DHA: Docosahexaenoic
FM: Fat mass
TBW: Total body water
ECW: Extracellular water
ICW: Intracellular water.
